# A Comprehensive Profiling System Integrating Myers-Briggs Type Indicator (MBTI) and Dominance, Influence, Steadiness, and Conscientiousness (DISC) for Personalized Health Training: Correlational Analysis and Usability Evaluation

**DOI:** 10.2196/73397

**Published:** 2025-07-18

**Authors:** Donghyun Kim, Dong Hun Lee, Mi Kyung Hwang

**Affiliations:** 1 Department of Industrial and Systems Engineering, Dongguk University, Seoul, Republic of Korea; 2 KOI Healthcare Co., Ltd., Seoul, Republic of Korea; 3 Department of Health and Medical Administration, Tongwon University, 268, Jungang-ro,Gwangju-si, Gyeonggi-do, 12735, Republic of Korea

**Keywords:** health behavior change, personalized programs, integrated profiling, personality types, Myers-Briggs Type Indicator, MBTI, Dominance, Influence, Steadiness, and Conscientiousness, DISC, personality assessment

## Abstract

**Background:**

This study proposes an integrated approach to developing personalized health behavior change programs by combining personality traits and behavior types. Existing tools, such as Myers-Briggs Type Indicator (MBTI) and Dominance, Influence, Steadiness, and Conscientiousness (DISC), have limitations: MBTI reflects internal tendencies but lacks behavioral insights, while DISC highlights behavior but overlooks deeper personality aspects. To address these gaps, the study integrates MBTI and DISC to create a comprehensive profiling system.

**Objective:**

The goal of this research is to design a novel profiling system that merges MBTI and DISC for personalized health management. This system aims to link personality traits with behavior patterns to enhance the effectiveness of tailored health behavior change programs.

**Methods:**

The study involved 3 phases: administering MBTI and DISC tests to 130 participants to analyze correlations, developing an integrated survey for health behavior analysis, and testing its usability with 20 experts for validation.

**Results:**

Significant correlations were observed between MBTI and DISC indicators, including a notable negative correlation between Thinking-Feeling (T/F) and Dominance (D), suggesting an inverse relationship between decision-making preferences and assertiveness. Usability testing results indicated high participant satisfaction, with an average SUS (System Usability Scale) score of 86.0. The SUS is a widely used questionnaire for measuring subjective assessments of usability. This score exceeded industry benchmarks for system usability. Expert evaluations further reinforced the system’s practical applicability, highlighting its potential to enhance user engagement through personalized behavioral insights.

**Conclusions:**

This study presents a combined MBTI and DISC profiling system, offering both theoretical insights and practical tools for health behavior change programs. Future research should validate its effectiveness with larger samples and explore broader applications in various health domains.

## Introduction

### Overview

In modern society, health behavior change has become increasingly important. According to the World Health Organization, adopting healthy lifestyle habits is essential for preventing chronic diseases such as obesity and cardiovascular disorders, as well as for improving overall quality of life [1,2]. While health-promoting activities, such as exercise and dietary management, can yield positive outcomes, standardized approaches that fail to account for individual personality traits and behavioral tendencies often face limitations in sustainability and effectiveness [3,4]. For instance, the same exercise program may feel burdensome to individuals with introverted tendencies but serve as a motivating factor for those with extroverted traits [5,6].

Several personality assessment models, including the Myers-Briggs Type Indicator (MBTI), Dominance, Influence, Steadiness, and Conscientiousness (DISC), Big Five, HEXACO, and Sixteen Personality Factor (16PF) Questionnaire, are widely used to analyze personality and behavior in fields such as psychology and health sciences. The MBTI categorizes personality into 16 types based on 4 dichotomies: Extraversion-Introversion, Sensing-Intuition, Thinking-Feeling, and Judging-Perceiving [7,8]. DISC focuses on 4 behavioral dimensions—Dominance, Influence, Steadiness, and Conscientiousness—emphasizing interpersonal interactions. The Big Five (OCEAN) assesses Openness, Conscientiousness, Extraversion, Agreeableness, and Neuroticism, with Conscientiousness and Neuroticism predicting health regimen adherence and emotional barriers. HEXACO adds Honesty-Humility to similar dimensions, offering insights into ethical health decisions. The 16PF Questionnaire measures 16 factors, such as emotional stability, refining health intervention strategies. The MBTI provides cognitive insights through its 16 personality types but lacks behavioral specificity. DISC focuses on 4 behavioral dimensions, Dominance, Influence, Steadiness, and Conscientiousness, but overlooks deeper psychological motivations. The Big Five measures broad traits such as Openness, Conscientiousness, Extraversion, Agreeableness, and Neuroticism, with Conscientiousness and Neuroticism predicting health regimen adherence and emotional barriers, yet it lacks detailed granularity. HEXACO adds Honesty-Humility, offering insights into ethical health decisions, but is less established. The 16PF Questionnaire refines health intervention strategies through 16 factors, such as emotional stability, but can be complex to administer.

We examined various analytical tools, and while each operates within its unique framework, no research has been identified that provides specialized solutions for complex and integrative domains, such as health behavior change programs [9].

This study proposes a novel approach that combines the strengths of MBTI and DISC to overcome these limitations. By integrating the analysis of individual personality traits and behavioral tendencies, the study aims to design personalized programs for health behavior change. Specifically, it seeks to identify correlations between the 2 tools and develop a methodology that maximizes the effectiveness of health behavior change programs, contributing both practical and academic value.

### Literature Review on MBTI and DISC and Their Integration

The MBTI, developed in the 1940s by Catherine Cook Briggs and Isabel Briggs Myers, is a psychological tool designed to understand and explain individual personality types [10]. It categorizes personalities into 16 types based on cognitive preferences and information processing styles, using 4 dichotomies: Extraversion (E)–Introversion (I), Sensing (S)–Intuition (N), Thinking (T)–Feeling (F), and Judging (J)–Perceiving (P). MBTI is widely used to understand interpersonal relationships, decision-making styles, and job performance [10]. However, its focus on static personality classifications can limit its ability to reflect situational flexibility [9].

DISC, developed from the theories of William Moulton Marston in the 1920s, analyzes behavioral styles based on 4 dimensions: Dominance (D), Influence (I), Steadiness (S), and Compliance (C). It is especially effective in understanding interpersonal dynamics, improving teamwork, and managing workplace interactions [11]. While DISC excels in analyzing external behavior and interaction patterns, it lacks the depth to fully capture internal psychological factors [12].

Although MBTI emphasizes psychological preferences and DISC focuses on external behaviors, both tools are valuable for understanding individual tendencies [13]. Integrating their strengths allows for a comprehensive understanding of both psychological and behavioral dimensions. This integrated approach is particularly suited for addressing complex issues such as health behavior change, where both internal and external factors play critical roles [14].

**Figure 1.**
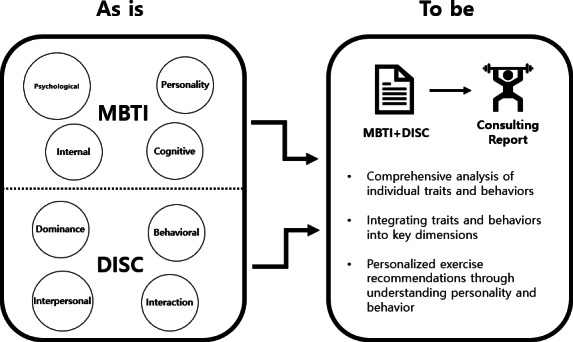
The methodology for generating consulting reports integrates insights from both MBTI and DISC frameworks. The MBTI model contributes psychological, internal, and personality-based dimensions (eg, personality and cognitive preferences), while the DISC model incorporates behavioral traits such as dominance, interaction styles, and action-oriented tendencies. DISC: Dominance, Influence, Steadiness, and Conscientiousness; MBTI: Myers-Briggs Type Indicator.

Prior to initiating this study, we recognized that the cognitive insights of the MBTI and the behavioral analysis of DISC are essential for health behavior change programs. However, no related studies combining MBTI and DISC to analyze health consulting or behavior change were identified. Accordingly, this study proposes an integration of the 2 models, aiming to analyze correlations between their core dimensions and develop a novel profiling system tailored for designing personalized health behavior change programs (Figure 1).

## Methods

### Verification of the Correlation Between MBTI and DISC

The purpose of this study is to systematically verify the correlation between MBTI and DISC by administering both surveys to the same group of participants. Through this process, the research aims to establish a foundation for an integrated understanding of personality and behavior and to provide fundamental data for developing an integrated personality analysis model.

The correlation analysis between MBTI and DISC centers on clearly identifying the similarities and differences that each tool provides and proposing a new approach that can leverage the strengths and compensate for the weaknesses of both tools. This study also explores the possibility of overcoming the limitations of existing personality assessment tools and developing a more efficient and practical personality profiling system.

For this research, 4 elements were set as the main criteria: interpersonal relationships, information-gathering style, decision-making style, and planning tendency. These criteria were selected to evaluate the potential applicability of this approach to health behavior change strategies, such as exercise and diet coaching. For example, the Dominance (D) type in DISC is predicted to have a high correlation with the Extroversion (E) and Thinking (T) traits in MBTI, suggesting that it may be suitable for goal-oriented methods. Meanwhile, the Influence (I) type is associated with the Feeling (F) trait, indicating a preference for a more friendly and motivational approach.

This study investigated the correlation between MBTI and DISC by surveying 130 Korean students. Demographic data (eg, age and gender) of the participants were collected to strengthen the validity of personality trait analysis, with detailed results summarized in Table 1.

**Table 1.** Demographic analysis of the survey subjects.

Variables and categories	Frequency, n (%)	
Year of birth		
2004-2000	100 (76.9)	
1999‐1990	19 (14.6)	
1989‐1980	11 (8.5)	
Total	130 (100)	
Sex		
Man	38 (29.2)	
Woman	92 (70.8)	
Total	130 (100)	

To analyze personality types using MBTI and DISC, each tool’s official survey was administered to ensure reliable responses from the students. Based on the survey results, this study examined the correlation between the 2 personality assessments and identified patterns in traits that appeared repeatedly. Graphs and charts were used to visualize these correlations and patterns, allowing for a clearer analysis of the relationship between MBTI and DISC.

### Refinement of an Integrated Trait Analysis Methodology and Proposed Survey Items

The aim of this study is to develop an integrated personality analysis model that concisely and practically uses personality traits by examining the correlations between the 4 DISC types and the 16 MBTI types.

DISC categorizes human behavioral styles into 4 types: Dominance (D) for goal-oriented and challenging individuals; Influence (I) for optimistic and social people; Steadiness (S) for calm and patient individuals; and Conscientiousness (C) for analytical and logical thinkers.

MBTI explains psychological preferences in perceiving and judging the world through 4 dichotomies. The ‘Attitude’ (Extraversion/Introversion) dichotomy relates to how we engage with others during exercise; for instance, questions gauge if one easily socializes in new exercise groups (Extraversion) or prefers solitary workouts for focus (Introversion). The “Perceiving Function” (Sensing/Intuition) focuses on how we gather information when choosing an exercise, assessing a preference for detailed rules (Sensing) versus imagining overall potential (Intuition). The “Judging Function” (Thinking/Feeling) examines how we make exercise decisions, involving questions about prioritizing objective data (Thinking) versus considering emotional satisfaction (Feeling). Finally, “Lifestyle” (Judging/Perceiving) looks at how we plan exercise, determining whether one strictly adheres to plans (Judging) or prefers flexible, spontaneous changes (Perceiving).

Based on the key indicators of these existing tools, this study will categorize core traits into 3‐8 types to design a model suitable for personalized coaching programs and behavior change strategies. We developed 40 survey items based on DISC and MBTI correlations, covering interpersonal relationships, information-gathering, decision-making, and planning tendencies, all adapted to specific contexts such as exercise and dieting. This survey will collect individual trait data, serving as a valuable source for designing customized health behavior programs.

### Customer Health Care Profiling Application Based on Survey Items

An expert group of 20 was selected for analysis. This sample was chosen to evaluate the practicality and validity of the integrated trait analysis model. Based on the survey results, a profiling system was developed to provide tailored health care advice and personality descriptions suited to each customer’s traits.

In developing the system, the 20 participants’ data were used to review the validity of the integrated model and 2 experts were consulted to assess its practicality.

This system can be used in various health care settings such as hospitals, health and fitness centers, and wellness counseling services, with an emphasis on delivering services, products, and personalized coaching optimized for each individual’s traits. For example, depending on the customer’s personality type, the system can propose effective exercise plans, diet methods, and stress management strategies, providing professional and customized health management solutions.

### Ethical Considerations

All information was collected anonymously. The data collected pertained to MBTI and DISC profiles, which are not individually identifiable and do not constitute sensitive personal information that could pinpoint individuals. At the time of the survey, participants were informed about the purpose of the study and their right to withdraw from participation at any time. Therefore, this study was deemed not to fall within the scope requiring institutional review board review. Specifically, this study was exempt from ethics review under Article 13(2) of the Enforcement Regulations of the Bioethics and Safety Act of Korea. This regulation exempts studies where researchers do not directly face identifiable individuals, and where “sensitive information” as defined by Article 23 of the Personal Information Protection Act is neither collected nor recorded.

## Results

### Analysis of the Correlation Between MBTI and DISC

**Figure 2.**
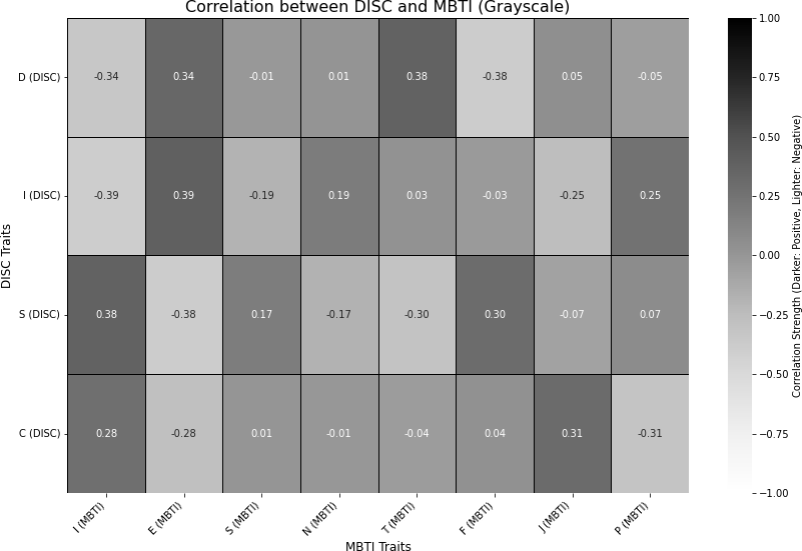
Summary of the analysis results of the correlation between MBTI and DISC, derived from the comparison table. Darker shades indicate stronger positive correlations and lighter shades represent stronger negative correlations. Only values with an absolute value of 0.15 or higher are displayed. DISC: Dominance, Influence, Steadiness, and Conscientiousness; MBTI: Myers-Briggs Type Indicator.

The correlation analysis results for identifying the relationship between the 2 personality assessment tools, MBTI and DISC, are presented in Figures2  3.

**Figure 3.**
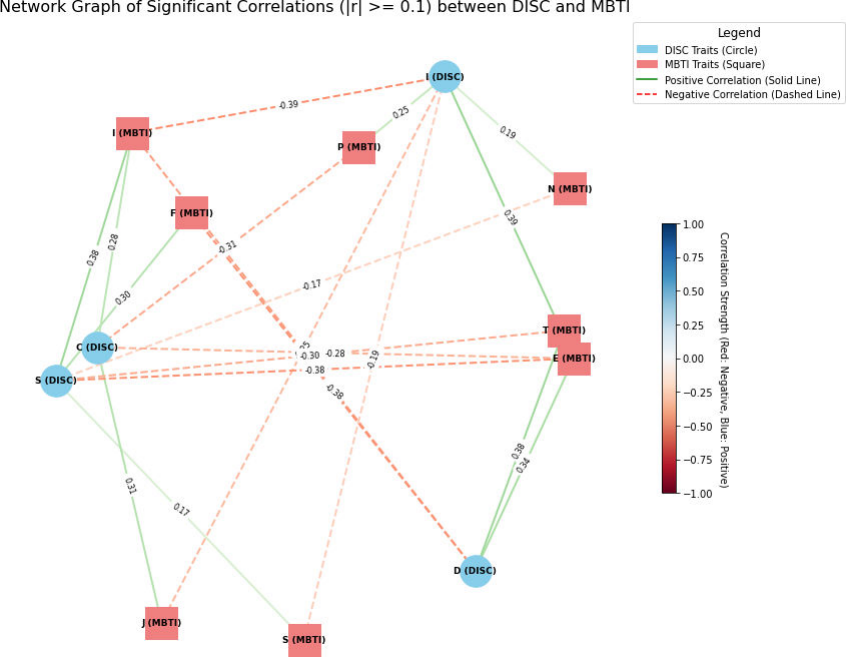
A network diagram of Myers-Briggs Type Indicator (MBTI)—comprising Extraversion (E)–Introversion (I), Sensing (S)–Intuition (N), Thinking (T)–Feeling (F), and Judging (J)–Perceiving (P)—and Dominance, Influence, Steadiness, and Conscientiousness (DISC) based on nodes and edges. Circles denote DISC and squares denote MBTI. Solid lines denote positive correlations and dashed lines denote negative correlations. Only correlations of 0.15 or higher were represented.

Through these findings, we aimed to determine the specific connections between MBTI’s personality type indicators (E/I, N/S, T/F, P/J) and DISC’s behavioral types (D, I, S, C), and to provide basic data for developing an integrated personality analysis model. The analysis was conducted using SPSS software (IBM Corp) and Pearson correlation coefficients were used to examine the relationships between the data. The sample consisted of 130 college and graduate students in their 20s and 30s.

First, both Extraversion (E) and Introversion (I) in MBTI showed significant correlations with all DISC types (D, I, S, C) (*P*<.001). Introversion (I) demonstrated positive relationships with Stability (S) and Conscientiousness (C), with a particularly stronger correlation with S (correlation coefficients 0.38, *P*<.001). In contrast, Extraversion (E) showed negative correlations with Dominance (D) and Influence (I), with a stronger negative correlation with I (correlation coefficients −0.38, *P*<.001).

Both Intuition (N) and Sensing (S) in MBTI showed significant correlations with Influence (I) and Stability (S) in DISC (*P*<.001). Intuition (N) had a weak negative correlation with Influence (I) (correlation coefficient −0.19, *P*=.03), whereas Sensing (S) had a weak positive correlation with Stability (S) (correlation coefficient 0.17, *P*<.04). Thinking (T) and Feeling (F) also exhibited significant relationships with Dominance (D) and Stability (S) in DISC (*P*<.001). Thinking (T) showed a strong negative correlation with Dominance (D) (correlation coefficient −0.38, *P*<.001), and Feeling (F) showed a positive correlation with Stability (S) (correlation coefficient 0.30, *P*<.001).

Finally, Perceiving (P) and Judging (J) in MBTI had significant correlations with Influence (I) and Conscientiousness (C) in DISC (*P*<.001). Perceiving (P) showed a negative relationship with Influence (I) (correlation coefficient −0.25, *P*<.001), while Judging (J) exhibited a positive relationship with Conscientiousness (C) (correlation coefficient 0.31, *P*<.001).

These results indicate that the correlation between MBTI and DISC is clearly evident in each type. For instance, according to the figure, MBTI’s Introversion (I) had a strong positive relationship with DISC’s Stability (S), while MBTI’s Thinking (T) showed a strong negative relationship with DISC’s Dominance (D). Despite MBTI and DISC each analyzing different aspects of personality—psychological preferences and behavioral patterns—these findings suggest that they can be mutually complementary.

### Proposing an Integrated Trait Analysis Model and Developing Survey Items

**Figure 4.**
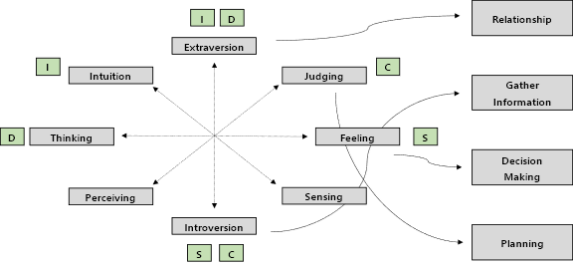
Type exploration and classification model based on MBTI-DISC. Considering the correlation between MBTI and DISC, 4 types (Relationship, Gather Information, Decision-Making, and Planning) were categorized. Clustering is represented using arrows. DISC: Dominance, Influence, Steadiness, and Conscientiousness; MBTI: Myers-Briggs Type Indicator.

The survey items were designed based on the DISC-MBTI correlation analysis results, narrowing down the classification into 4 types. A model was developed to categorize individuals into these 4 types, and the survey items were structured accordingly as Figure 4.

Based on the designed survey items, exercise prescriptions have been summarized in Table 2.

**Table 2.** Exercise prescription types based on the Myers-Briggs Type Indicator (MBTI)–Dominance, Influence, Steadiness, and Conscientiousness (DISC) survey. The table systematically classifies exercise prescription types by organizing them into key categories, including type, description, recommended exercises, and coaching strategies.

Categories and types	Description	Recommended exercises	Coaching, support, and planning strategies
Relationship			
Group-oriented	Finds motivation in activities with others, prefers social interaction.	Group fitness (Zumba, aerobics), team sports	Provide positive feedback, enhance collaboration with peers.
Individual-oriented	Prefers solo activities, focuses on achieving personal goals.	Solo running, gym equipment exercises	Emphasize individual performance, provide an independent environment.
Expert-dependent	Requires guidance and structured plans from trainers or experts, prefers systematic feedback.	Personal training, customized exercise plans	Offer professional, data-driven feedback.
Competition-oriented	Gains motivation through competition or performance comparison, values winning.	Competition-based sports (tennis, boxing, swimming)	Visualize performance comparisons with rivals, provide rewards for victories.
Gather information			
Experience-seeker	Chooses exercises based on reviews or others’ experiences.	Trendy programs, popular classes	Provide review-based materials, emphasize user reviews and ratings.
Principle-seeker	Logically selects exercises based on scientific evidence and principles.	Systematic, structured exercise programs	Explain exercise effects and provide scientific data, offer professional resources.
Decision-making			
Logical goal-oriented	Sets specific goals and approaches exercise systematically to achieve them.	HIIT^a^, strength training	Set performance-based goals, motivate with concrete data.
Emotion or relationship-oriented	Values emotional satisfaction or building relationships, prioritizes fun and experience.	Dance classes, group sports	Provide emotional support, foster positive relationship building.
Planning			
Structured or detail-oriented	Consistently follows a systematic and detailed plan.	Regular yoga, Pilates	Provide detailed schedules, set step-by-step goals.
Flexible and people-oriented	Adjusts plans flexibly according to circumstances, prefers interaction with others.	Trekking, diverse activity experiences	Emphasize autonomy, offer engaging and appealing options.

a HIIT: high-intensity interval training.

Based on the DISC-MBTI correlation analysis, the survey items were designed to narrow the classification down to 4 types. A model was then developed to categorize individuals into these 4 types, and corresponding survey items were created accordingly. Finally, the exercise prescriptions derived from these survey items are summarized.

In this study, we developed survey items to classify exercise tendencies by measuring individuals’ relational tendencies. Each item assesses aspects such as the individual’s level of activity, expression styles, and relationship preferences in interpersonal contexts. This approach identifies preferred environments and motivational factors during exercise. For the purposes of this study, we focused exclusively on methods for categorizing relationships with others during exercise.

### Implementation and Validation of an Exercise Prescription Reporting Service

**Figure 5.**
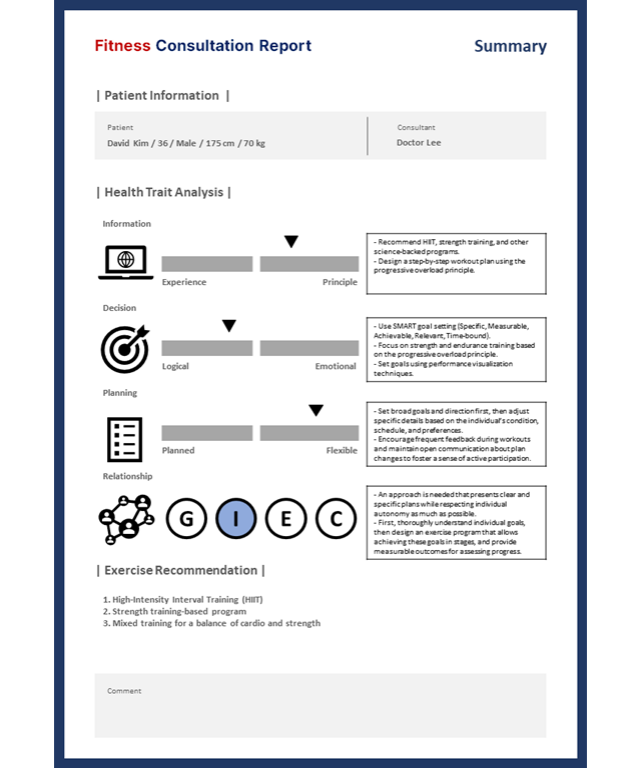
Example of an exercise prescription consulting report for 4 categories: relationship, information-gathering, decision-making, and planning.

A practical profiling web application was implemented to input survey items and verify whether the results matched actual patient information. The web application was developed based on Flask and calculated the total scores by summing up the scores for each of the 4 survey items. The results of this process are presented in Figure 5.

A pilot test was conducted to validate the practicality and reliability of the proposed integrated trait analysis model. This process assessed whether the proposed profiling system effectively analyzed individuals’ personality and behavioral traits and whether it could be practically used. The validation process consisted of a pilot test, result analysis and interpretation, and a practicality evaluation through expert interviews.

The pilot test involved applying the profiling system, developed based on MBTI and DISC survey data, to a group of 20 experts. The experts responded to the survey items, and the system analyzed their personality and behavioral types to generate customized profiling results. This process confirmed whether the profiling system could accurately classify and interpret types based on the survey data.

### System Usability Scale Score Result for Prescription Reporting Service

To evaluate the usability of the proposed system, a System Usability Scale (SUS) assessment was conducted with 20 experts, as Table 3.

**Table 3.** Survey participants by occupation. The survey was primarily conducted with trainers, physical therapists, and professors.

Occupation	Participants, n
Trainer	8
Physical therapist	4
Exercise specialist	4
Professor in related fields	3
Aerobics instructor	1

**Figure 6.**
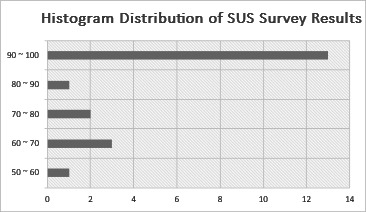
Histogram distribution of System Usability Scale (SUS) survey results. The majority of scores clustered in the 90‐100 range, although a few respondents provided lower ratings.

The SUS, originally developed by Brooke [15], is a widely used and reliable tool for measuring system usability across various domains. The questionnaire consists of 10 items rated on a 5-point Likert scale, designed to assess both positive and negative aspects of usability. Odd-numbered items measure positive usability attributes (eg, ease of use and system integration), while even-numbered items measure negative usability attributes (eg, complexity and inconsistency). The standard scoring methodology was applied, where responses to odd-numbered items were adjusted by subtracting 1, and even-numbered items were adjusted by subtracting the response from 5. The final SUS score was computed by summing the adjusted values and multiplying by 2.5, following the established methodology described by Lewis and Sauro [16]. The resulting SUS score of 86.0 indicates a high level of usability and user satisfaction, as shown in Figure 6.

Trainers, physical therapists, exercise specialists, professors in related fields, and aerobics instructors responded positively, highlighting that the system is intuitive, effectively integrates various functions, and is easy to navigate. In addition, participants across clinical, academic, and fitness backgrounds stressed that a highly personalized approach, tailored to individual user characteristics, is key to enhancing engagement and usability. Some experts, particularly trainers and exercise specialists, noted that the system has the potential to serve as an effective tool for continuous health care management by adapting to users’ needs. However, some participants emphasized the need for more detailed exercise coaching programs and suggested conducting further real-world validation to ensure the system’s effectiveness in practical settings. These findings, supported by feedback from professionals across diverse fields, including trainers, sports dance instructors, and professors, confirm the system’s practical applicability and its potential for real-world implementation.

## Discussion

### Principal Results

This study identified the integrated correlation between MBTI and DISC, addressing the limitations of existing personality theories and enabling theoretical expansion. MBTI focuses on internal psychological preferences, while DISC emphasizes external behavioral patterns. By combining the strengths of these 2 tools, this study proposed a new analytical framework for comprehensively understanding individuals’ personality and behavior.

The integrated trait analysis model, based on the correlation between MBTI and DISC, clarified the relationship between personality and behavioral types and validated its theoretical soundness through data-driven analysis. This research provides a new direction for personality analysis and behavior prediction studies in academic fields and serves as foundational data for future research.

In practical applications, this model can be used as a valuable tool for health care and health behavior change programs. For example, personalized health programs can be designed by optimizing exercise plans, diet strategies, and stress management techniques according to individual personality and behavioral types. For instance, individuals with DISC’s Stability (S) and MBTI’s Introversion (I) may benefit from quiet, structured exercise environments, whereas those with DISC’s Influence (I) and MBTI’s Extraversion (E) may thrive in sociable and dynamic activities.

In addition, the findings contribute to the development of personalized coaching programs that motivate behavior change based on a deep understanding of individual tendencies and behavior patterns. This system can be applied in diverse practical environments, such as hospitals, fitness centers, and counseling institutions, offering high user satisfaction while enhancing service quality.

### Limitations

This study offers valuable insights into the correlation between MBTI and DISC, but it has several limitations. First, our sample size was limited to 130 Korean college and graduate students in their 20s and 30s, which may restrict the generalizability of our findings. The sample was also somewhat skewed toward female students and those born in the 2000s. The reliance on self-reported survey data also introduces potential biases, such as social desirability or self-perception. Future research should include larger, more diverse populations and incorporate additional data collection methods, like observational studies or third-party assessments.

We recognize that the cultural context of South Korea significantly influenced our survey results, particularly among the 20s demographic, which is sensitive to social norms. For example, Korea’s collectivistic culture may modulate the expression of Extraversion (MBTI) and emphasize diligence for Conscientiousness (DISC C-type) or harmony for Steadiness (DISC S-type) in responses.

Furthermore, our study focused solely on MBTI and DISC. Integrating additional frameworks, such as the Big Five Personality Traits, could offer a more comprehensive understanding. The practical applications discussed, like healthcare and behavior change programs, also require further real-world validation to confirm their effectiveness. Finally, we simplified complex traits into 4 main categories for usability, potentially overlooking nuanced individual differences and not accounting for longitudinal changes in personality. Future studies should explore these dynamics and validate the profiling system in broader technological and organizational contexts.

### Conclusions

In conclusion, this study successfully identified the integrated correlation between MBTI and DISC, combining their strengths to propose a novel analytical framework for understanding personality and behavior. By validating the integrated trait analysis model with data-driven methods, the research highlighted its potential to clarify the relationship between psychological preferences and behavioral patterns. This model offers both theoretical contributions to personality analysis and practical applications, such as personalized health behavior programs, which can optimize exercise plans, diet strategies, and stress management based on individual traits. While further validation in diverse settings is needed, the findings pave the way for future innovation in health care, counseling, and other fields that benefit from tailored behavior change strategies.

## Abbreviations

16PF: Sixteen Personality Factor
DISC: Dominance, Influence, Steadiness, and Conscientiousness
MBTI: Myers-Briggs Type Indicator
SUS: System Usability Scale
